# The Effect on Length of Stay After Implementation of Discharging Low Acuity Patients From Triage

**DOI:** 10.7759/cureus.17640

**Published:** 2021-09-01

**Authors:** Tommy Y Kim, Connor Ohmart, Zara Khan, Michael Lance, Steven Kim

**Affiliations:** 1 Emergency Medicine, HCA Healthcare, Riverside Community Hospital, Riverside, USA

**Keywords:** emergency medicine, metrics, length of stay, triage, quality improvement

## Abstract

Introduction

Overcrowding in the emergency department is a complex and challenging issue across the nation. The increasing number of patients seeking care in the emergency department leads to overcrowding and therefore decreased available rooms and slower throughput. As part of a quality improvement project to improve throughput, we implemented a policy encouraging the discharge of non-emergent patients directly from triage.

Methods

This was a retrospective pre- vs post-implementation analysis of a discharge process from triage to decrease emergency department length of stay. We implemented a policy that allowed the physician assistant to discharge lower acuity patients directly from triage. We collected daily length of stay metrics for a two-week period prior to and a two-week period after the implementation of the policy. Total and daily pre- and post-implementation length of stay means were compared and reported.

Results

There was a total of 1044 (pre-implementation) and 1063 (post-implementation) patients evaluated during the study period. There was a significant mean difference improvement in the overall length of stay post-implementation of 18.43 minutes (95% CI, 15.45 - 21.40). When comparing the differences for the day of the week, all days showed a statistically significant mean improvement in the length of stay of greater than 10%.

Conclusion

Discharging low acuity patients directly from triage can lead to a reduction in length of stay. Future studies are needed to determine the impact of different confounders on the length of stay of patients who are discharged from triage, as well as studies to evaluate the outcomes of patients that have been discharged from triage.

## Introduction

Overcrowding in the emergency department (ED) is a complex and challenging issue felt by EDs across the nation. The increasing number of patients seeking care in the ED leads to overcrowding and therefore decreased available rooms and slower ED throughput [1]. This, in turn, leads to delays in patient evaluation and is associated with longer lengths of stay (LOS), more left without being seen (LWBS) frequencies, poor patient satisfaction, and increased patient morbidity and mortality [2-3]. In fact, studies show that patients with longer waiting room times believe that they receive inferior care [4-6]. Therefore, it comes with no surprise that emergency medicine clinicians and hospital administrators continue to search processes that will improve ED throughput for the increasing ED patient population.

Multiple techniques have been employed to combat the issue of improving ED throughput, including the formation of Rapid Medical Assessment (RMA) teams and the utilization of mid-level providers or triage liaison providers. However, the impact of such interventions on LOS and the proportion of patients LWBS has been mixed in prior analyses [3,7-8].

As part of a quality improvement (QI) project to improve ED throughput, we implemented a policy encouraging discharge of non-emergent patients directly from triage. We report the results of this intervention on the effects of LOS after the implementation of this policy.

## Materials and methods

This was a retrospective pre- vs post-implementation analysis of a QI project implementing discharge processes from triage to decrease ED LOS. The ED is a community-based emergency medicine residency program with a census of approximately 120,000 patient visits per year. There is a dedicated fast track (FT) unit within the ED that utilizes the same initial triage and waiting areas as the main ED.

We implemented a policy that allowed the physician assistant (PA), who resides in the triage area 24 hours a day, to discharge all Emergency Severity Index (ESI) 5 patients and select ESI 4 patients. ESI 5 patients are considered non-urgent and required no ED resources. ESI 4 patients are considered less urgent and required only one resource for a disposition to be reached. Prior to the implementation of the discharge from triage policy, we had an established triage RMA process. Patients were assigned an ESI level by the triage nurse based on chief complaint, vital signs, comorbidities, and acuity of illness. The triage PA performed a rapid assessment, initiated orders, and patients would be assigned an ESI level. ESI 4 and 5 patients who required few to no resources with stable vital signs were all assigned to the FT area of the ED.

The revised discharge from triage policy was implemented on May 1, 2019. We collected data on the LOS of all ESI 4 and 5 patients before and after the implementation of the discharge from triage protocol. We evaluated the average LOS for a two-week period before and a two-week period after implementing this process to determine if there was a significant change in the LOS of patients seen in the FT. We determined a priori that a 10% difference would be considered significant to keep this policy in place after the completion of the QI project. The LOS was defined as the difference between the arrival time and departure time.

The data were collected from time stamps and patient disposition data that are recorded in our electronic medical record for all patient encounters. We collected daily LOS metrics for a two-week period from April 1 to April 14 prior to the implementation of the policy as our control group and then collected daily LOS metrics for a two-week period from May 13 to May 26 after the implementation of the policy as our study group.

Data were analyzed using the STATA SE 14.0 (Stata Corporation, College Station, Texas) statistical software. Total and daily pre- and post-implementation LOS means were compared using a two-sample t-test with equal variances. A P-value less than 0.05 was considered to be significant. Sample size calculations were based on mean LOS for ESI 4 and 5 patients with standard deviation values for our own patient population prior to implementation of the discharge from triage policy. Our power analysis showed that a sample size of 253 patients in each group would have a power of 0.80 to detect a LOS difference of 10% between the two groups with a two-tailed significance of 0.05. This QI project was classified as exempt by our research oversight committee and institutional review board.

## Results

There was a total of 1044 (pre-implementation) and 1063 (post-implementation) patients with either ESI 4 or 5 seen in the two-week pre- and post-implementation period. The combined ESI 4 and 5 pre-intervention had a mean LOS of 87.61 minutes (95% CI, 85.46 - 89.77) compared to the post-intervention mean LOS of 69.19 minutes (95% CI, 67.14 - 71.24). There was a significant mean difference improvement in the overall LOS of 18.43 minutes (95% CI, 15.45 - 21.40), p < 0.001. The subgroup analysis evaluating ESI 4 and 5 patients separately found that the ESI 4 had a pre-intervention mean of 88.92 minutes (95% CI, 86.74 - 91.11) and a post-intervention mean of 75.81 minutes (95% CI, 73.57 - 78.04) for an overall improvement of 13.12 minutes (95% CI, 9.99 - 16.25), p < 0.001. ESI 5 patients showed an even greater improvement with a pre-intervention mean of 63.08 minutes (95% CI, 53.52 - 72.63) and post-intervention mean of 40.09 minutes (95% CI, 37.65 - 42.52), with a mean difference of 22.99 minutes (95% CI, 16.24-29.73), p < 0.001. When comparing the differences for the day of the week, all days showed a statistically significant mean improvement in the LOS of greater than 10%, which is shown in Table 1.

**Table 1:** Length of Stay for Total Group by Day of Week

Day of Week	Mean LOS (95% CI)	
Sunday (Pre)	82.96 (78.31 – 87.62)	
Sunday (Post)	74.41 (68.94 – 79.87)	
Mean difference	8.55 (1.38 – 15.73)	P = 0.020
Monday (Pre)	97.57 (91.36 – 103.78)	
Monday (Post)	74.07 (69.11 – 79.04)	
Mean difference	23.50 (15.60 – 31.40)	P = <0.001
Tuesday (Pre)	88.50 (83.51 – 93.48)	
Tuesday (Post)	70.22 (64.97 – 75.47)	
Mean difference	18.27 (11.07 – 25.48)	P = <0.001
Wednesday (Pre)	90.82 (84.62 – 97.01)	
Wednesday (Post)	58.68 (53.81 – 63.55)	
Mean difference	32.14 (24.31 – 39.96)	P = <0.001
Thursday (Pre)	77.06 (71.04 – 83.08)	
Thursday (Post)	67.98 (62.20 – 73.77)	
Mean difference	9.07 (0.77 – 17.38)	P = 0.032
Friday (Pre)	87.20 (81.22 – 93.18)	
Friday (Post)	64.92 (59.25 – 70.58)	
Mean difference	22.28 (14.07 – 30.50)	P = <0.001
Saturday (Pre)	86.81 (81.26 – 92.37)	
Saturday (Post)	71.64 (65.98 – 77.31)	
Mean difference	15.17 (7.24 – 23.10)	P = <0.001

## Discussion

ED overcrowding is a system-wide problem with no simple or immediate solutions. Most of the literature on improving efficiency in the ED is based on changes such as incorporating RMA teams and adding advanced practice providers or even attending physicians to the triage area [7-10]. The use of advanced practice providers to manage ESI 4 and 5 patients is not a novel concept, as many centers use this strategy [2]. However, having the PAs in our triage area provides a more in-depth assessment of ESI 4 and 5 patients to more accurately identify candidates who could be appropriately treated and discharged prior to being placed in our FT area is novel. There are no known previous studies on the discharge of low acuity patients directly from triage and how that directly affects the overall LOS for these patients. While the use of PAs to discharge low acuity patients directly from triage may not address the leading causes of overcrowding in the ED, our study suggests a positive influence on an important factor - LOS. In fact, LOS for patients with an ESI of 4 or 5 decreased by 18.43 minutes. The findings of this study suggest that implementation of this type of intervention could provide some improvement in flow in a high-volume ED and decrease overcrowding by increasing throughput and getting patients out of the ED faster. More importantly, since LOS declined for this population, existing resources can be used more efficiently.

The following limitations should be acknowledged when interpreting the results of this study. First, a variable number of nurses work in the ED on a day-to-day basis, and therefore the effect of nurse-to-patient ratio and how that affects patient LOS was not factored. But there were no departmental changes on nurse staffing with the implementation of the discharge process. Furthermore, while triage nurses have general guidelines to follow when assigning ESI levels, these are often subjective and therefore possibly affected our study. Lastly, while the goal was to have PAs discharge ESI 5 and select ESI 4 patients directly from triage, it is unclear how many of these patients were sent to FT or the main ED for further care lengthening their LOS.

## Conclusions

Our results indicate that changes made to the disposition of low acuity patients can significantly improve their overall ED LOS. The ED LOS decreased by an average of 18.43 minutes (21% improvement) after the implementation of a discharge from triage policy, and each day of the week showed a clinically and statistically significant decrease in the LOS of low acuity patients. We conclude that the use of a policy utilizing a PA discharging low acuity patients directly from triage leads to an overall reduction in the LOS of these patients. Future studies are needed to determine the impact of different confounders on the LOS of patients who are discharged from triage, as well as studies to evaluate the outcomes of patients that have been discharged from triage.
